# Modulation of Host-Pathogen Communication by Extracellular Vesicles (EVs) of the Protozoan Parasite *Leishmania*

**DOI:** 10.3389/fcimb.2019.00100

**Published:** 2019-04-11

**Authors:** George Dong, Alonso Lira Filho, Martin Olivier

**Affiliations:** Infectious Diseases and Immunity in Global Heath Program, The Research Institute of the McGill University Health Centre, Montreal, QC, Canada

**Keywords:** *Leishmania*, macrophage, exosome, host-pathogen interaction, immunomodulation

## Abstract

*Leishmania* genus protozoan parasites have developed various strategies to overcome host cell protective mechanisms favoring their survival and propagation. Recent findings in the field propose a new player in this infectious strategy, the *Leishmania* exosomes. Exosomes are eukaryotic extracellular vesicles essential to cell communication in various biological contexts. In fact, there have been an increasing number of reports over the last 10 years regarding the role of protozoan parasite exosomes, *Leishmania* exosomes included, in their capacity to favor infection and propagation within their hosts. In this review, we will discuss the latest findings regarding *Leishmania* exosome function during infectious conditions with a strong focus on *Leishmania*-host interaction from a mammalian perspective. We also compare the immunomodulatory properties of *Leishmania* exosomes to other parasite exosomes, demonstrating the conserved, important role that exosomes play during parasite infection.

## Introduction

Leishmaniasis is a complex pattern of diseases caused by sand fly-transmitted *Leishmania sp*. With more than 300 million people living in *Leishmani*a-endemic areas (Alvar et al., 2012), there are over 2 million new cases of leishmaniasis every year resulting in 30 000 deaths each year (WHO, 2015). In mammals, *Leishmania* parasites establish a persistent infection by inducing macrophage dysfunction through direct manipulation of macrophage signaling. We have deciphered the mechanisms whereby *Leishmania* exploits macrophage signaling pathways to block microbicidal functions and innate inflammatory responses during infection (Olivier et al., 2005; Isnard et al., 2012). Our lab has previously demonstrated how *Leishmania* major surface protease GP63 manipulates macrophage responses to promote infection through direct activation of protein tyrosine phosphatases (Gomez et al., 2009), thus negatively downregulating JAK and MAP kinase pathways and cleaving key signaling molecules such as the transcription factors AP-1 and NF-κB (Abu-Dayyeh et al., 2008; Contreras et al., 2010; Shio et al., 2015).

Additional investigation from our lab has demonstrated how *Leishmania* parasites, like the majority of eukaryotic cells and other protozoan parasites, release extracellular vesicles (EVs) that play a key role in macrophage modulation. These cellular entities are a vehicle for biologically active macromolecules, such as proteins and nucleic acids, which, once delivered, act on the physiology and function of host cells. Generally grouped according to various criteria including size, density, and location within cells, these vesicles originate from the cell membrane. They have been termed microparticles, microvesicles, or even ectosomes. On the other hand, exosomes are produced inside and released by multivesicular bodies (MVB) when the latter merge with the plasma membrane (Tkach and Thery, 2016). In scientific literature, the term “exosome” is generally used in reference to a group of mixed EVs regardless of their intracellular provenance. Advanced scientific techniques should soon be capable of distinguishing the many types of EVs (Thery et al., 2009; Raposo and Stoorvogel, 2013; Tkach and Thery, 2016). Recently, the universality of exosomes and their numerous possible applications in medicine (diagnostics or treatment) have garnered them special consideration.

40–120 nm in size, ultracentrifugation (at 100,000 g or greater) is necessary to the sedimentation of exosomes (Johnstone et al., 1987). Linear sucrose gradients can be used for additional purification given their specific density in the medium (1.13–1.19 g/ml) (Raposo et al., 1996). Exosomes occur when the endosomal membrane invaginates into MVBs. Melding of the latter with the plasma membrane (PM) leads to exosome secretion (Thery et al., 2002). This novel EV biogenesis pathway was first noted in transferrin secretion by reticulocytes (Harding et al., 1983; Pan et al., 1985). This distinguished exosomes from other EVs that were thought to simply bud from the PM. The mechanism of protein sorting into these vesicles is remarkably organized as well as contingent on the type and biological status of the original cell (Thery et al., 2002). That said, given their frequent enrichment within exosomes, select proteins are thought to be necessary to exosome generation in MVBs. This suggests some conservation in terms of the sorting and biogenesis pathway (Baietti et al., 2012).

Exosomes originating from distinct cell varieties consist of different endosome-associated proteins. Rab GTPases are one example, or proteins included in MVB synthesis (Alix, Tsg101) (Thery et al., 2009; Taylor and Gercel-Taylor, 2011; Raposo and Stoorvogel, 2013). Additionally, within exosomes there are numerous categories of proteins that are constantly present: heat shock proteins (HSP60, HSP70, HSP90), proteins with adhesion activity (tetraspanins CD81, CD63, CD37), annexins (I, II, V, VI), cytoskeletal proteins (actin, tubulin), metabolic enzymes, and proteins with translational (Elongation Factors 1, 2) or signaling activity (Schorey and Bhatnagar, 2008; Simpson et al., 2008; Thery et al., 2009; Silverman et al., 2010a; Hassani et al., 2011; Taylor and Gercel-Taylor, 2011; Yang and Robbins, 2011; Raposo and Stoorvogel, 2013; Atayde et al., 2015). Exosomes also contain mRNAs and microRNAs (miRNA) that can be transmitted to cells of interest in a functional state (Valadi et al., 2007; Zomer et al., 2010).

## The Critical Role of Exosomes in Cell-Cell Communication

Exosomes play a role in numerous biological processes, both pathophysiological and physiological. They have been identified in many types of biological material including saliva, urine, plasma, breast milk, and amniotic fluid (Admyre et al., 2007; Keller et al., 2007, 2011; Looze et al., 2009; Moon et al., 2011). Currently, uses for exosomes in the treatment of cancer and infectious diseases are being extensively studied, as well as their possible function in regenerative therapy, targeted therapy, and more (Lener et al., 2015). With the popularity of exosome research, the International Society for Extracellular Vesicles (ISEV) has implemented a gold standard for the isolation and analysis of extracellular vesicles, exosomes included. For instance, this includes the use of nanovesicle tracking assay (NTA) for the determination of population homogeneity, use of transmission electron microscopy (TEM) for morphological observation, use of linear sucrose gradients to assess their density, as well as the use of mass spectrometry, western blotting, and high throughput sequencing to precisely analyze their protein and RNA contents (Lötvall et al., 2014). Classification of *Leishmania* exosomes requires such analyses.

The enrichment of certain molecules in exosomes, including tetraspanins and integrins (involved in adhesion and cell communication), suggests that the cooperation between said proteins and their counterparts found on the plasma membrane of the target cell facilitates exosome delivery. Moreover, various stimuli can induce changes in tetraspanin composition that impacts the selection of targets by exosomes (Rana and Zoller, 2011; Andreu and Yanez-Mo, 2014). Exosomes merge with the plasma membrane of the target cell for direct cargo transfer (Silverman et al., 2010a) or undergo endocytosis or phagocytosis (Feng et al., 2010; Bastos-Amador et al., 2012). Additionally, certain exosomes can deliver their information through simple attachment to target cells (fusion, endocytosis, or phagocytosis are unnecessary); specifically exosomes expressing MHC II in their interaction with T-cells (Yang and Robbins, 2011). That said, demonstration of the majority of the aforementioned methods of interaction came from experiments performed *in vitro*; as such, the mechanisms taking place *in vivo* are still in question.

## Secretion of Exosomes Containing *Leishmania* Proteins

As with higher eukaryotes, the parasite *Leishmania* and members of the trypanosomatids use the ER/Golgi-mediated secretion system (McConville et al., 2002; Corrales et al., 2010), polarizing parasite proteins toward the parasites' flagellar pocket (Field et al., 2007) Several *Leishmania* virulence factors, including the metalloprotease GP63 and other immunomodulatory proteins, use this pathway to exit the host cell (Yao et al., 2003; Joshi et al., 2005). For example, the cysteine proteases of *L. mexicana* are sorted to lysosomes and then released via the flagellar pocket upon their passage into the Golgi apparatus (Brooks et al., 2000).

One of the first pieces of evidence for the use of non-conventional secretory mechanisms in trypanosomatids was revealed while studying their hydrophilic acylated surface proteins (HSAPs). HSAPB is a surface protein found in many *Leishmania*, possessing the unique characteristic of lacking a signal peptide, a transmembrane domain, and a GPI-anchor site. Denny et al. revealed a sequence of amino acids in the protein's N-terminal region that seems to act as a signal peptide, allowing HSAPB distribution to the plasma membrane. To support this, they transferred a fluorescent GFP protein to the parasite surface by simply adding this sequence of amino acids. Interestingly, HSAPB transfection of mammalian cells also brought about its cell surface translocation, establishing that similar protein trafficking can occur in higher eukaryotes (Denny et al., 2000).

Included among non-conventional mechanisms of protein secretion is the exosomal pathway, since the majority of exosome proteins do not bear a predicted signal peptide (Thery et al., 2009; Hassani et al., 2011; Atayde et al., 2015). Importantly, this pathway is not rare and found to be a cardinal mechanism utilized by a great number of eukaryotic organisms, including protozoan parasites such as *Leishmania*.

The accurate analysis of the complex proteins of exosomes is now possible by advanced mass spectrometry. This further applies to the identification of a myriad of proteins belonging to the secretomes of various cell types (Skalnikova et al., 2011), microscopic unicellular (Silverman et al., 2008; Cuervo et al., 2009; Atyame Nten et al., 2010; Geiger et al., 2010) and multicellular organisms (Moreno et al., 2011), and various tissues (Pardo et al., 2012). Information stemming from these analyses is of paramount importance for our understanding of the mechanisms of secretion and responses of cells to diverse stimuli. Notably, different laboratories have reported that, similar to higher eukaryotes, the majority of *Leishmania* species studied to date also have a low percentage of exosomal proteins that bear a signal peptide (Thery et al., 2009; Hassani et al., 2011; Atayde et al., 2015). This suggests that a great majority of proteins belonging to the secretome of various organisms are non-conventionally secreted (Silverman et al., 2008; Cuervo et al., 2009; Atyame Nten et al., 2010; Geiger et al., 2010).

Vesicle release, common to organisms including prokaryotes, protozoans, fungi, archaea, and higher eukaryotes, has been proposed as universal (Deatherage and Cookson, 2012). Exosomes are special in that they are derived from the endocytic pathway, rather than from direct budding from the plasma membrane like other EVs (Tkach and Thery, 2016). In mammalian cells, the most important pathway requires the action of endosomal sorting complexes necessary for transport (ESCRT) proteins. These ESCRT proteins were first discovered in yeast by selecting for mutant yeasts deficient in vacuole biogenesis and sorting, resulting in the discovery of vacuolar sorting proteins (VPS) (Banta et al., 1988). There are 4 ESCRT complexes, ESCRT 0-III, and they are responsible for the formation of MVBs, as well as the sequestration of ubiquitinated proteins into the intraluminal vesicles (ILVs) (Raposo and Stoorvogel, 2013).

Interestingly, this ESCRT machinery seems common to eukaryotes, including trypanosomatids (Leung et al., 2008). In trypanosomatids, the exact processes responsible for secretion of exosomes are still unclear, but they appear comparable biochemically to those of mammalian exosomes in terms of density and morphology (Silverman et al., 2008, 2010a; Trocoli Torrecilhas et al., 2009). Furthermore, TEM analyses offer convincing arguments for the direct, *in vivo* secretion of exosomes by *Leishmania* through MVBs (Atayde et al., 2015), while Rab GTPases, Alix, and ESCRT orthologs were found through proteomic analyses of *Leishmania* exosomes/exoproteome (Silverman et al., 2008, 2010a; Corrales et al., 2010; Deatherage and Cookson, 2012; Atayde et al., 2015); this suggests a pathway analogous to that of the mammalian ESCRT-dependent pathway previously reported. Although ESCRT-independent exosome biogenesis pathways have been described in mammalian cells and other parasites, this area has yet to be explored in depth in *Leishmania* (Theos et al., 2006; Trajkovic et al., 2008). For example, sphingomyelinase and tetraspanin CD63 have been identified in *Fasciola hepatica*, the common liver fluke, which represent an alternative pathway for cargo sorting and invagination of the endosome for MVB formation (Cwiklinski et al., 2015). The mechanisms for exosome secretion itself remain unclear, though soluble NSF-attachment protein receptor (SNARE) complexes and the RAB family of small GTPases have been suggested to be involved in mammalian cells (Thery et al., 2009; Raposo and Stoorvogel, 2013).

## *Leishmania* Exosomes: Its Impact on Cutaneous Leishmaniasis Progression

In the last 10 years, several groups reported that various *Leishmania* species secrete exosomes in culture and in the midgut of its sand fly vector. These vesicles are actively manipulating host signaling and immune cell functions, as per the enrichment by *Leishmania* exosomes of virulence factors such as GP63 (Silverman et al., 2010a,b; Hassani et al., 2014; Atayde et al., 2015). Experiments performed on macrophages *in vitro* and with mice *in vivo* brought clear evidences that enrichment of *Leishmania* virulence factors by exosomes is of cardinal importance for the infectious process and the development of pathologies related to leishmaniasis.

**Figure 1:**
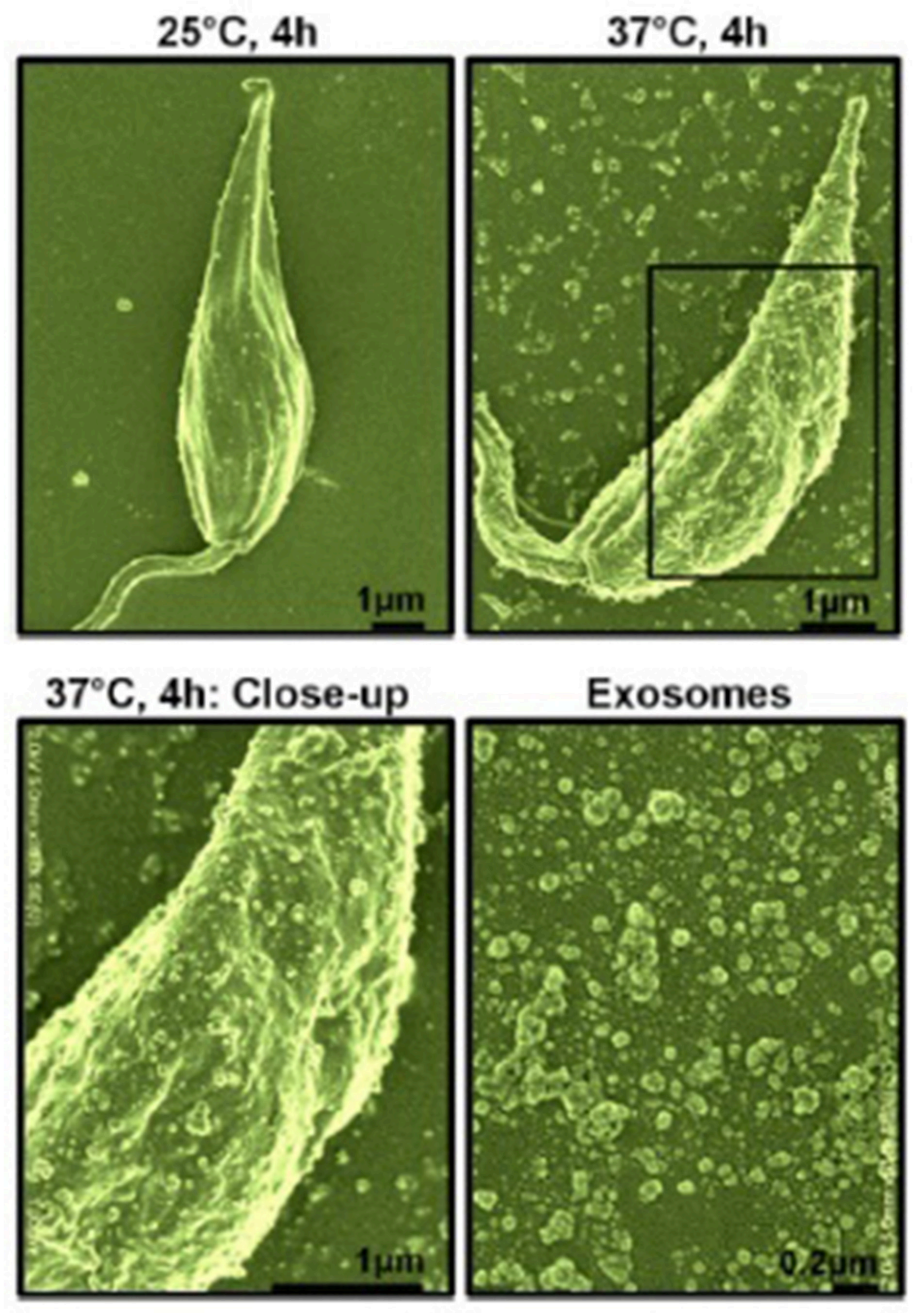
Scanning electron microscopy of *L. mexicana* parasites before and after temperature shift. Top left, Parasites at 25°C for 4 h; Top right, Parasites at 37°C for 4 h; Bottom left, Close-up of parasite surface at 37°C; Bottom right, purified exosomes. Exosomes were of 40–100 nm in size as per electron microscopy observation [from Hassani et al. (2011)].

Our interest, in regards to the study of *Leishmania* exosomes, was initially triggered by the observation of intra-macrophage vesicles clustering around the *Leishmania* surface protease GP63, leading us to hypothesis that *Leishmania* parasite can form and release extracellular vesicles, including exosomes (Gomez et al., 2009; Gomez and Olivier, 2010). Initial evidence for *Leishmania* exosome secretion was obtained through the study of *L. mexicana* exoproteome (Hassani et al., 2011), but Silverman et al. were the first to report *bona fide* secretion of *Leishmania* exosomes (Silverman et al., 2010a). However, in our study, we found that temperature shift (TS) mimicking the conditions for inoculation of *Leishmania* into its host was sufficient to cause a rapid and important augmentation in protein release from the *Leishmania* parasite in culture alongside a clear increase of exosome-like vesicles being released from the parasites' surface (see Figure 1). Additionally, the majority of *Leishmania* exosomal proteins were found to be secreted non-conventionally, as per their proteomic analysis (Hassani et al., 2011). We further showed that *L. mexicana* exosomes released upon TS possessed a similar capacity to inhibit several macrophage microbicidal functions as the parasite *per se*, relying on the induction of PTP activity concurring to the alteration of key host cell signaling pathways.

Thereafter, the role of exosome-enriched *Leishmania* GP63 and its impact on immune cell functions was explored. Using a *L. major* gp63^−/−^ (KO), it was found that the immunomodulatory capacities of leishmanial exosomes deficient in the metalloprotease GP63 were strikingly abrogated in comparison to *L. major* wild type (WT). This strongly supports the cardinal role of exosome-enriched *Leishmania* virulence factors in the infectious process. Using qRT-PCR analysis, this was further confirmed by the divergent capacity of WT and KO exosomes to induce macrophage gene expression, such as cytokines and chemokines. Of utmost interest is how the proteomic analysis of WT and KO exosomes revealed such drastic modification of their protein contents, suggesting that GP63 in *Leishmania* participates in the regulation of exosomal protein sorting (Hassani et al., 2014).

Studies performed in Reiner's lab further established the impact of *Leishmania* exosomes on host immune responses (Silverman et al., 2010a,b). For instance, they observed that exosomes from *Leishmania donovani* can modify the secretion of IL-10 and TNF-α by human monocytes subjected to IFN-γ stimulation. Furthermore, they found that mice treated with *L. donovani* exosomes will have an augmented production of CD4^+^ T-cells producing IL-10 and IL-4 once challenged with infectious *Leishmania*, which could explain in part the exacerbated skin inflammation they observed (Silverman et al., 2010b). Findings stemming from these studies suggested that *Leishmania* exosomes are mainly favoring an immunosuppressive status permitting the parasite to better propagate within its infected host. More recently, a study by Lambertz et al. reported enrichment of small RNAs originating from non-coding RNAs in various *Leishmania* species' exosomes. Unfortunately, the role for this cargo has not been investigated in depth, therefore its potential impact in the infectious process remains uncertain (Lambertz et al., 2015).

## Release of *Leishmania* Exosomes within Sand Fly Midguts

**Figure 2:**
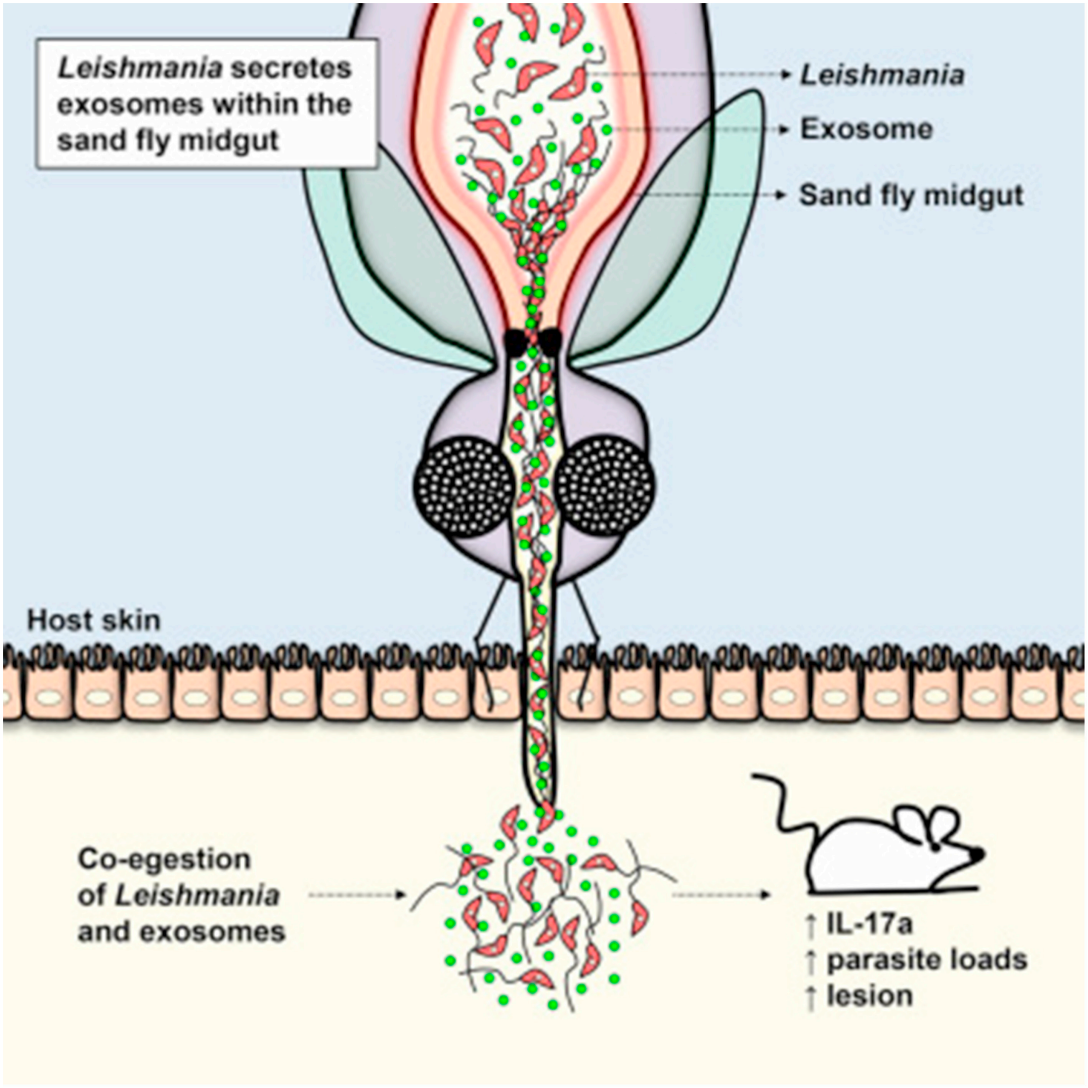
Cartoon depicting the release of *Leishmania* exosomes within the sand fly midguts and their egestion during the insect blood meal. Their co-inoculation seems to favor skin hyperinflammation and increase in parasitic load [from Atayde et al. (2015)].

For many years, a great number of investigations dealing with extracellular vesicles were done with vesicles obtained from diverse biological fluids and supernatants from cells cultured *in vitro*. Until recently, the observation of exosome biogenesis and their exit from the cell in an *in vivo* context has proved to be an incredible challenge. After years of effort, we have been able to report a seminal finding demonstrating that *Leishmania* exosomes were produced and released in the sandfly vector midgut and are egested during blood meals together with *Leishmania* parasites (see Figure 2). This co-inoculation was found to significantly augment skin lesion due to the synthesis of key pro-inflammatory cytokines, such as IL-17a (Atayde et al., 2015). Of utmost importance, this work represents the first demonstration that GP63-enriched *Leishmania* exosomes are critical vector-inoculated virulence factors and solidly places these *Leishmania* vesicles as important infectious agents necessary for proper progression of the *Leishmania* life cycle.

Contrasting with the work of Silverman et al. using *L. major* exosomes (Silverman et al., 2010b), we found the induction of IL-17a to be increased relative to IL-4 (Atayde et al., 2015). This can be in part due to the fact that instead of vaccinating mice with exosomes, we directly co-injected exosomes and parasites together, therefore better mimicking what is happening in natural conditions. IL-17a is known to be a hallmark of neutrophil recruitment during the development of *Leishmania*-induced human and murine lesions (Lopez Kostka et al., 2009; Boaventura et al., 2010). Previous findings from our laboratory are in accordance with the fact that *Leishmania* exosome inoculation trigger neutrophil recruitment at the site of injection (Deatherage and Cookson, 2012).

## *Leishmania* RNA Virus 1 Enhances Mucocutaneous Leishmaniasis Using Exosomes

*Leishmania*, being eukaryotic organisms themselves, are a host to infectious agents as well, including viruses like *Leishmania* RNA Virus 1 (LRV1) (Guilbride et al., 1992). The significance of this was revealed when Ives et al. reported that LRV1 modulates mucocutaneous leishmaniasis when investigating *L. guyanensis*, a member of the *L. Viannia* subgenus and a common cause of mucocutaneous leishmaniasis (Ives et al., 2011). They identified two groups of *L. guyanensis* clones, metastatic (LgM^+^) and non-metastatic (LgM^−^), based on their ability to cause secondary lesion formation in hamsters. When investigating the role of macrophage Toll-like receptors (TLR), they found that metastasis caused by LgM^+^ was dependent on TLR3 and enhanced by TLR7. This was particularly interesting since TLR3 and TLR7 recognize double stranded and single stranded RNA, respectively (Doyle and O'Neill, 2006), implicating a viral infection taking place in the LgM^+^ infected macrophages. Ives et al. were able to quantify LRV1 infection of *L. guyanensis* by RT-qPCR, and showed that LgM^+^ had higher viral load compared to LgM^−^. They further demonstrated that TLR3^−/−^ and TLR7^−/−^ mice did not display enhanced inflammation or pathology when infected with LgM^+^ compared to LgM^−^ and LgLRV^−^.

Building upon this discovery, our group recently demonstrated that *Leishmania* exosomes play an important role in the LRV1 life cycle by protecting the whole LRV1 virion from a potential dangerous external environment (e.g., from the action of RNAses) (Atayde et al., 2019). Another defense mechanism that this exosomal coating confers to the virus is the ability to disguise LRV1 since these *Leishmania* exosomes are naturally integrated into the naive recipient parasites, leading to an increase of in infectivity. In this way, *Leishmania* exosomes act both as a protecting and facilitating viral carrier. Exosomes bear a striking resemblance to viral particles on many levels, including their structure and physical properties. This similarity is a possible reason why exosomes are exploited by HIV to facilitate their distribution (Teow et al., 2016). In fact, a variety of viruses, including HCV, HAV, HSV, and EBV, have all had exosome release in the course of infection linked to their pathological processes (Meckes and Raab-Traub, 2011; Alenquer and Amorim, 2015). However, this is the first time exosomes have been shown to carry the whole virion. We could also conclude that the newly LRV1-infected parasites generated a more aggressive form of leishmaniasis in a mice infection model, demonstrating how this exosomal coating process used by LRV1 is also important in the context of the mammalian host; it can be said that *Leishmania* and LRV1 have a mutualistic relationship facilitated by exosomes.

## Parasite-Derived Exosomes/EVs

The prospective uses of exosomes and various pathogen-secreted vesicles as immunomodulators are countless; they are being further explored for their potential in treatments or vaccine development. Combined with cutting-edge genomics techniques like CRISPR, a better understanding of pathogen-derived EVs may allow the engineering of exosomes and other EVs that stimulate and promote an immune response and host protection in the hopes of combatting infectious diseases.

Exosomes have been shown to possess both immune-stimulatory and -inhibitory effects in eukaryote pathogens. For example, Oliveira et al. recently demonstrated that *Cryptococcus neoformans* produces exosome-like vesicles that lead to the release of TNF, IL-10, and NO through stimulation of macrophages. Macrophage priming with the aforementioned vesicles facilitates the killing of fungi (Oliveira et al., 2010). In contrast, extracellular vesicles originating from *T. cruzi* and administered to mice led to a worsened infection; lower iNOS and higher IL-4 and IL-10 levels were observed, as well as greater localization of parasites to the viscera and heart (Trocoli Torrecilhas et al., 2009).

In addition, another parasite of the genus *Trypanosoma, T. brucei*, has been reported to use extracellular vesicles in their pathogenesis. In the study performed by Szempruch et al., bloodstream parasites could be observed producing extracellular vesicles enriched in flagellar proteins and virulence factors including serum resistance-associated protein (SRA), a well-defined protein of this group (Trocoli Torrecilhas et al., 2009). More interestingly, when stimulating non-human infectious trypanosomes with these vesicles, SRA was transferred to these parasites, giving them the ability to evade the innate immune response. Finally, these extracellular vesicles were able to fuse with erythrocytes and make them express Variant Surface Glycoprotein (VSG), leading to a rapid clearance of these cells by the immune system and resulting in anemia in two distinct mouse strains. In a more recent study by another group, these vesicles were also found to affect the social motility of parasites; they drove parasites away, repelling them from overstressed or compromised cells, thus providing a novel function for these vesicles in the parasite life cycle (Eliaz et al., 2017).

In a similar fashion, *Trichomonas vaginalis* exosomes were found to be produced and released with similar biophysical properties to mammalian vesicles, sharing many proteins found in the mammalian exosome proteome (e.g., tetraspanins, Alix, and Rabs) (Twu et al., 2013). In addition to their ability to fuse and deliver proteins to vaginal epithelial cells, exosomes from strains of very adherent parasites improved the adherence of strains of less adherent parasites. Furthermore, the extracted exosomes led to production of IL-6 in stimulated epithelial cells while down-regulating IL-8 yield. This potential immune modulation was further studied by Olmos-Ortiz et al. (2017), who showed *T. vaginalis* exosomes not only increased IL-6 production but highly increased IL-10 production in macrophages. The possible anti-inflammatory role of *T. vaginalis* exosomes was tested in a murine *in-vivo* model, which showed that pre-treatment with these vesicles lead to a diminished inflammatory response after *T. vaginalis* infection, favoring the persistence and viability of the parasite.

## Impact of Immune Cell Exosomes and Control over Infectious Agents

The function of host exosomes during infection by related parasites has also been explored during this time period. While most in-depth studies were related to cancer, reports of exosomes playing unique, crucial roles during host viral, bacterial, or protozoan infection are accumulating.

Schorey lab reported that exosomes containing glycopeptidolipids derived from *Mycobacterium* can be released from macrophages infected by various species of the bacteria, and contain numerous *Mycobacterium*-derived proteins (Bhatnagar and Schorey, 2007; Giri et al., 2010). Particularly, it was demonstrated that said exosomes can lead to protection against infection by *M. tuberculosis* in mice through production of iNOS and TNF-α by naive macrophages; a pro-inflammatory response (Bhatnagar and Schorey, 2007; Cheng and Schorey, 2013). Further testing by said group explored infection of macrophages with *Mycobacterium* ssp., *Salmonella typhimurium*, and *Toxoplasma gondii* (protozoan parasite) and demonstrated that the produced exosomes also prompted MyD88- and TLR-dependent production of TNF-α by naive macrophages (Bhatnagar et al., 2007). Interestingly, exosomes from dendritic cells infected with *T. gondii* used to vaccinate mouse fetuses were reported to offer protection against congenital infection (Beauvillain et al., 2009).

Infection of reticulocytes by *Plasmodium yoelii* and immunization with the released exosomes was investigated by Martin-Jaular et al. Said exosomes had previously been shown to incorporate the parasites' proteins, and authors noted impressive protection of immunized mice when later challenged with infection by *P. yoelii* (Martin-Jaular et al., 2011). Research from 2013 demonstrated how *P. falciparum*-infected erythrocytes are capable of using released exosomes to communicate with certain parasites among a population, evidence suggesting that exosomes may be crucial to the transfer of *P. falciparum* to its insect vector (Regev-Rudzki et al., 2011). Likewise, a newer investigation performed by the aforementioned researchers showed that these vesicles contain small RNA and genomic DNA of the parasite and can reach human monocytes, which will lead to a STING-dependent DNA sensing, possibly acting as an immune decoy, favoring parasite survival (Sisquella et al., 2017).

In this same time period, we studied the outcome of infection by *Leishmania* on release of exosomes by macrophages (Hassani and Olivier, 2013). Exosome production by macrophages (untreated, LPS-stimulated, and *L. mexicana*-infected) was compared using proteomic analyses. We noted that stimulation by LPS and infection by *Leishmania* lead to both similar and dissimilar variations in protein function group sorting (particularly plasma membrane-associated proteins) into exosomes; additionally, signaling molecules (including MAPK) were differentially induced in naive macrophages by said exosomes. This work demonstrated that, within exosomes liberated by infected macrophages, *Leishmania* GP63 was the sole enriched *Leishmania* protein (Hassani and Olivier, 2013).

Importantly, from the perspective of all the work performed up to now, it is clear that *Leishmania* exosomes are pro-active components of this parasitic infection, both *in vitro* and *in vivo*, mainly influencing the early innate immune response during *Leishmania* infection to favor the parasite's survival, allowing it to fully establish itself within the mammalian host. Although it has been suggested that non-vesicular components display immunomodulatory potential (Perez-Cabezas et al., 2019), offering the counterpoint that EVs are not the sole effector of immunomodulation, the vast majority of findings stemming from these various studies fully establish *Leishmania* exosomes as cardinal virulence factors.

In conclusion, we hope that this review brings about a new and more in-depth understanding of the part that *Leishmania* exosomes and various infectious agents play in the context of host-parasite interactions, with a particular focus on the establishment of infection. Future research in this field of investigations is critical for the development of new vaccine and diagnostic tools.

## Author Contributions

The first draft was done by MO. AF and GD added new information.

### Conflict of Interest Statement

The authors declare that the research was conducted in the absence of any commercial or financial relationships that could be construed as a potential conflict of interest.
